# Intensive Care Unit Room Characteristics and Association with the Development of Delirium

**DOI:** 10.3390/neurosci7030061

**Published:** 2026-05-20

**Authors:** Sharon Nguyen, Bilal Khoncarly, Timothy N. Holbrook, Simran Demla, Xuan Wang, Leslie Rodriguez, Lavanya Srinivasan, Alexander Bastidas, Jennifer A. Walker

**Affiliations:** 1 Department of Medicine, Baylor Scott & White All Saints Medical Center, Fort Worth, TX 76104, USA; sharon.nguyen@bswhealth.org (S.N.); bilal.khoncarly@bswhealth.org (B.K.); timothy.holbrook@bswhealth.org (T.N.H.); simran.demla@bswhealth.org (S.D.); lavanya.srinivasan@bswhealth.org (L.S.); alexander.bastidas@bswhealth.org (A.B.); 2 Department of Research Analytics, Baylor Scott & White Research Institute, Dallas, TX 75204, USA; xuan.wang@bswhealth.org; 3 Department of Graduate Medical Education, Baylor Scott & White All Saints Medical Center, Fort Worth, TX 76104, USA; leslie.rodriguez@bswhealth.org; 4 Department of Emergency Medicine, Baylor Scott & White All Saints Medical Center, Fort Worth, TX 76104, USA; 5 Anne Burnett Marion School of Medicine, Texas Christian University, Fort Worth, TX 76104, USA

**Keywords:** delirium, critical illness, ICU environment, ICU room design, CAM-ICU, hospital length of stay, circadian rhythm

## Abstract

Introduction: Delirium is associated with worse outcomes in critically ill patients. Factors that could reduce delirium are patients’ environment and maintaining circadian cycles, but whether certain room characteristics improve the incidence of delirium is unclear. Our objective was to investigate whether the presence of windows or doors in patients’ rooms is associated with lower rates of delirium. Methods: In this retrospective, cohort study, adult, medical patients admitted to the Intensive Care Unit (ICU) between 1 January 2024 and 1 July 2024 were identified. Clinical and room characteristics were collected. The primary outcome was the development of Confusion Assessment Method for the Intensive Care Unit (CAM-ICU) positive status. Secondary outcomes included ICU and hospital length of stay (LOS) and mortality. Results: Four hundred fifty-eight (458) patients met inclusion criteria (mean age 61.7 +/− 16.4 years; 51.3% male). In the adjusted multivariate analysis, neither the presence of windows (aOR 2.2; 95% CI 0.7–6.2; *p* = 0.155) nor closed-format rooms (aOR 0.1; 95% CI 0.0–1.2; *p* = 0.077) were significantly associated with CAM-ICU positivity. Although an initial association was observed between the presence of windows and increased hospital LOS (aMR 1.4; 95% CI 1.1–1.8; *p* = 0.008), this did not maintain statistical significance after False Discovery Rate (FDR) correction (q > 0.05). No significant associations were found for other secondary outcomes, including ICU mortality or ICU LOS. Conclusion: Room characteristics were not significantly associated with delirium or secondary outcomes after FDR correction. However, given the study’s limited power to detect moderate differences, these findings do not definitively rule out an architectural influence on delirium and warrant further investigation in larger cohorts. Studies with more discrete measurement of room characteristics are needed to investigate these associations further.

## 1. Introduction

Delirium is a frequent condition in critically ill patients, affecting 60–80% of ventilated patients, and is associated with worse outcomes [1]. Patients experiencing delirium have longer days on mechanical ventilation (MV), increased length of stay (LOS), chronic cognitive dysfunction, and increased mortality [2,3,4,5]. Because of these detrimental effects, researchers and Intensive Care Unit (ICU) teams have worked to understand the epidemiology and pathophysiology of delirium as well as to find ways to reduce its prevalence.

Preventing delirium is more effective than treating delirium [6]. In fact, despite efforts to utilize anti-psychotics and other pharmacotherapy [7,8], there is no consistently effective treatment for delirium. In contrast, preventing delirium includes instituting measures such as in the Pain, Agitation/Sedation, Delirium, Immobility, and Sleep disruption (PADIS) guidelines: recognizing and treating pain, early mobility and liberation from the ventilator, choosing medications wisely, and creating the dynamic of family engagement [9]. Bundled approaches appear to mitigate delirium prevalence in a dose-dependent fashion [1,10,11]. ICU teams often work to optimize environments, providing quiet times and promoting quality sleep; however, different environmental features of ICU rooms such as whether they have a window or a closed door are less understood [12,13].

Despite efforts to understand deliriogenic environments, there are mixed results regarding the qualities of ICU rooms and associated risk of developing delirium. Keep et al. performed early work comparing critically ill patients admitted to rooms with no window or with a non-transparent window and found markers of decreased memory and attention, concluding that construction of hospitals with windowless units was unacceptable [14,15]. Kohn et al. conducted a similar study and paradoxically found no difference in the rates of delirium or mortality [16]. Meanwhile, studies that look at multi-patient and single-patient rooms have similarly conflicting results, with Caruso et al. describing a lower prevalence of delirium in patients in single-bed compared to multi-bed rooms, but Zaal et al. showing no change in the prevalence of delirium between cohorts but a decrease in the number of days with delirium in patients in single-bed rooms [2,17]. Our study aims to evaluate multiple room attributes and assess whether patients exposed to certain characteristics have an associated higher occurrence of delirium. Specifically, we hypothesized that open-format (without a door) and windowless rooms will have a higher incidence of delirium. By observing the impact of room types and possible associations with delirium, we hope to add to the existing literature.

## 2. Materials and Methods

### 2.1. Study Design, Setting and Patient Selection

We conducted a retrospective cohort study of patients in a multi-unit intensive care department in an urban, tertiary care center from 1 January 2024 to 1 July 2024. This ICU has a mixed population of both surgical and medical patients, with approximately 3000 annual admissions. The ICUs are divided into three geographic units across a single floor, and occasionally, the post-operative care unit is used as an overflow for ICU patients. The rooms can be closed-format with a door and four fixed walls, or open-format with curtain dividers as three of the walls and another curtain instead of a door. Rooms are single-windowed or windowless. Room types are mixed within these three geographical units, which have 8–16 beds. To reduce confounding factors, we included only non-surgical patients. These non-surgical patients are attended by one team of medical intensivists, with two intensivists covering per day from 7 a.m. to 7 p.m., and two nurse practitioners covering from 7 p.m. to 7 a.m. When medical patients are admitted to the ICU, they are admitted to rooms on a first-come, first-serve basis.

Delirium is assessed using the Confusion Assessment Method for the ICU (CAM-ICU), which is an externally validated tool that is performed by our nursing staff once per 12 h shift [18,19]. The CAM-ICU consists of four components assessing acute change or fluctuation in mental status, inattention, disorganized thinking, or altered level of consciousness. To utilize the CAM-ICU score, patients must have a Richmond Agitation Sedation Scale (RASS) equal to or greater than −3. If the patient’s score is less than −3, then the CAM-ICU is documented as “unable to assess” (UTA).

The study protocol was reviewed and approved by the investigators’ Institutional Review Board (024-170) on 28 February 2024. The study met criteria for expedited review, and no formal consent was required due to the observational nature of this study. Prior to initiation of this study, educational information was provided by investigators and nursing educators to medical staff, residents, and nursing staff regarding CAM-ICU scoring to increase compliance with documentation.

Patients were included in this analysis if they were older than 18 years and were admitted to the ICU during the study period. Patients who were admitted for medical conditions were eligible. Excluded patients were patients with ICU stays of less than 24 h, surgical patients, patients with missing data, and patients that changed rooms. Patients with stays of less than 24 h were excluded from the study to avoid including extremes of illness, such as too well to need the ICU or too sick to survive the initial day. Surgical patients were excluded from this study to reduce confounding between ICU care team practices. Patients who changed rooms during the study were also excluded due to difficulties tracking timing of room changes, and therefore, timing of room characteristic exposures. We excluded patients with missing demographic data, room information, or Acute Physiology And Chronic Health Evaluation (APACHE) IV mortality risk scores because the lack of complete data prevented a standardized comparison. Our initial cohort of patient encounters to review was extracted by our hospital system’s data analytics dashboard by searching for adult, medical patients admitted during our timeframe. Data analytics utilized hospital account records to evaluate whether surgery occurred in a main operating room or ambulatory surgical center during that stay, and these records were filtered out prior to handoff to the investigative team.

The primary outcome for this study was the number of patients experiencing a positive CAM-ICU score. Secondary outcomes included ICU LOS, hospital LOS, disposition, and mortality. The composite of CAM-ICU positive combined plus “CAM-ICU Unable to Assess” was included as an exploratory outcome. We collected demographic data including age, sex, race, and language spoken. Admission diagnoses, whether patients were intubated, days of MV, whether sedation or antipsychotics were used, ICU LOS, hospital LOS, and discharge disposition including mortality were collected. Baseline illness severity was assessed using the APACHE IV predicted mortality risk score, expressed as a percentage (0–100%) calculated based on the worst physiologic parameters within the first 24 h of ICU admission. The mortality risk score was directly recorded from the electronic medical record. Length of time on MV was calculated by adding up all consecutive days of MV and rounding to the nearest day. We also recorded, in a similar manner, the number of days patients received sedation, if relevant; however, RASS data were not obtained due to inability to track specific RASS score timing in relation to important outcomes such as CAM-ICU scores. Patients’ room numbers were collected to cross reference with the recorded room characteristics. Missing data regarding if a patient’s room was moved or not moved were imputed as “not moved”.

Prior to the study period, room characteristics were independently recorded by two investigators surveying each room; characteristics included room number and the presence of a door with containment by four walls and a door compared to rooms with no door, with separation between rooms by curtains. The presence or lack of a window was also recorded. Other information that was recorded was whether there was a view of the window from the bed, whether the window was obstructed from the outside, and if the room was near a loud, automatic door. Ultimately, only the exposures of open/closed format and no window/window were included in our analysis due to low prevalence of other characteristics. Rooms were defined as “with a window” or “windowless” and “closed-format” to describe four walls and a door or “open-format” to describe rooms separated with curtains and no door present.

Data were extracted into a standardized Microsoft Excel spreadsheet (Microsoft Corp., Seattle, WA, USA) by primary investigators (B.K., T.H., S.D., S.N.) using the electronic medical record, EPIC (Verona, WI, USA).

### 2.2. Statistical Analysis

We did not perform a priori sample size calculation due to the retrospective nature of this study. Continuous variables were evaluated for normality using visual histograms and the Shapiro–Wilk test. Normally distributed variables were described with the mean (standard deviation), and non-normally distributed variables were described with the median (interquartile range). Student’s *t*-test (for normally distributed variables) and non-parametric Wilcoxon Mann–Whitney tests (for non-normally distributed variables) were utilized to compare between independent groups or continuous variables, respectively. Categorical variables were described in numbers (percentages) and compared by Pearson’s chi-square test or Fisher Exact test. Outcomes were also analyzed using multivariate analyses with generalized linear mixed models, including Logistic regression and Gamma regression for binary outcomes and the LOS outcomes, respectively. Window presence and door format were modeled as main effects simultaneously in the primary models. To evaluate whether the association between window presence and the outcome differed by room closed format, we tested a window-by-door interaction term using likelihood ratio test (LRT) based on nested primary multivariate models. Diagnosis and ICU type were incorporated into the primary models as fixed effects, and unique room ID was modeled as a random intercept. Predicted mortality risk score was included in the primary multivariate models as a covariate to account for differences in baseline illness severity. The APACHE IV predicted mortality risk score was modeled using a natural cubic spline with 3 degrees of freedom (ns(APACHE IV, df = 3)) to allow for potential non-linear effects. Secondary multivariable mixed models were adjusted not only for all the variables in the primary models such as window, door, mortality risk score, diagnosis and ICU types, but also for MV, sedation exposure, and restraint use. An alternative modeling approach was used in which logistic regression models were fitted without a random intercept, and room-level cluster-robust standard errors were applied. Gamma regression with log link was utilized to model the right-skewed hospital LOS outcome, while adjusting for the same selection of fixed effect.

Logistic mixed model fitting was assessed using goodness–of-fit statistics, random intercept variance, and inspection of residuals. Gamma mixed model fitting was assessed using random intercept variance and simulation-based residual diagnostics (DHARMa).

Sensitivity analyses were performed with generalized linear models (Logistic and Gamma) on all the samples, excluding PACU samples, and excluding UTA samples, respectively.

Odds ratios (OR) with 95% confidence interval (CI) were used to estimate the association between binary outcomes and patient groups. Mean ratios (MR) with 95% CI were used for estimating the effects of patient groups on the continuous LOS outcomes. Two-tailed *p*-values < 0.05 were considered statistically significant. To account for multiple comparisons, we applied the Benjamini–Hochberg false discovery rate (FDR) correction across multiple comparisons. A post hoc power analysis was conducted for the primary delirium outcome, comparing CAM-ICU positivity between patients in windowless versus windowed rooms using the observed group sizes (72 vs. 386), a two-sided α of 0.05, and the observed event rates (26.4% vs. 19.4%), which corresponded to an approximate OR of 1.49. Power was estimated using a two-sample comparison of proportions with unequal group sizes. Data were analyzed using R software (version R.4.4.0; Vienna, Austria).

## 3. Results

### 3.1. Patient Characteristics

**Figure 1:**
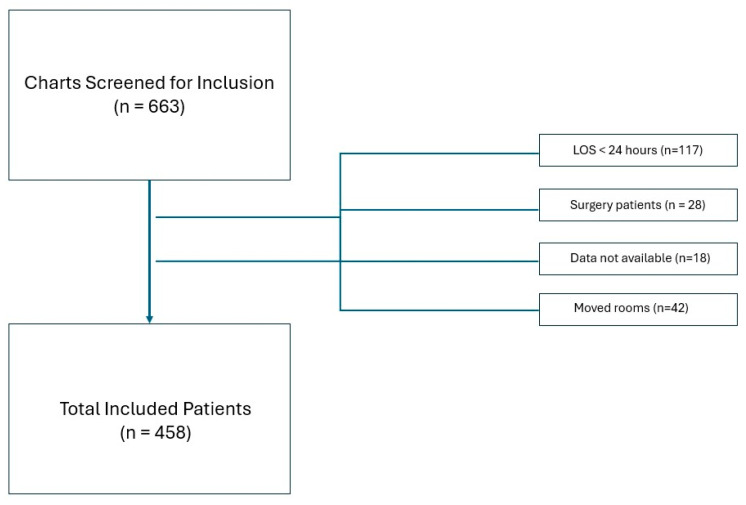
Flow Diagram of Patient Selection.

Six hundred sixty-three patients (663) were identified through our hospital dashboard, and 458 were included in the final analysis (Figure 1). The most common reason for exclusion was LOS < 24 h (*n* = 117), followed by patients that moved rooms (*n* = 42). There were 18 patients from the initial extraction that had missing data, including eight with APACHE IV predicted mortality risk scores not available and 10 with other demographic and room data missing.

Three hundred eighty-six patients (84.3%) were in rooms with windows, and 72 (15.7%) were in windowless rooms. One hundred twelve patients (24.5%) were in open-format rooms, while 346 (75.5%) were in rooms with doors. Baseline demographics were similar across age, sex, and race (Table 1). In our included cohort, mean age (SD) was 61.7 (16.4), with 51.3% male. Most patients were white (76.9%) and spoke English as their primary language (90.4%). One hundred forty-six (31.9%) patients were on MV, and the median with interquartile range (IQR) predicted mortality score was 10.4 (4.6, 27.2). Patients in rooms with a window had a significantly higher median predicted mortality score of 11.1 (IQR (5, 29.8), *p* = 0.022), rate of MV (34.2%), and presence of sedation (38.1%) compared to patients in windowless rooms. Likewise, patients in closed-format rooms had a significantly higher predicted mortality score of 11.4 (IQR 5.2, 30.2, *p* = 0.015) and rate of MV (34.4%) compared to those in open-format rooms. Further demographics and baseline clinical characteristics of patients can be found in Table 1.

### 3.2. Room Characteristics

The findings of our room survey can be found in Appendix A. Unit levels are divided into a mixed ICU (ICU), medical/surgical ICU (MSICU), cardiovascular ICU (CVICU), and post-anesthesia care unit (PACU). A total of 44 patient rooms were evaluated across the four unit levels: ICU (*n* = 16), CVICU (*n* = 15), MSICU (*n* = 8), and PACU (*n* = 5). Overall, 35 rooms (79.5%) had windows, while nine rooms (20.5%) did not. Fifteen (34%) did not have a view of the window from the bed. Closed-format rooms with doors accounted for 32 rooms (72.7%), whereas 12 rooms (27.3%) were open-format without doors. All CVICU and ICU beds had a door, while seven of eight (87.5%) MSICU beds lacked a door. Doorless rooms used curtains for privacy but did not have the noise reduction benefits of walls or doors. All 16 ICU beds had windows, with the window visible from the patient’s bed. Fourteen of 15 (93%) CVICU beds had windows, and most windows (86.7%) were visible from the patient’s bed. Windows were present in five MSICU rooms (62.5%), though none had window visibility from the patient’s bed. Two CVICU rooms (13.3%) and two MICU rooms (25%) were located near an automatic unit entry door. All PACU rooms were open format without doors, and none had windows or window visibility from the patient’s bed. No PACU rooms were located near automatic doors.

### 3.3. Primary Outcome: Incidence of CAM-ICU Positive Scores

A total of 116 (25.3%) patients experienced delirium. In rooms with windows, 102 (26.4%) patients experienced delirium compared to 14 (19.4%) patients who did not have a window. No missingness was encountered in the multivariate logistic mixed regression that models the binary incidence of CAM-ICU positive scores. The primary model converged successfully, and scaled residuals were examined and did not indicate extreme deviations. Random effect variance of the random intercept of room was estimated to be 0.14. The interaction term between window presence and door format was not statistically significant; therefore, the primary models retained window presence and door format as main-effect terms. APACHE IV predicted mortality score was modeled by natural cubic spline with 3 degrees of freedom (ns(APACHE IV, df = 3)), suggested by the LRT test’s significance (*p* = 0.013). Due to the low value of estimated random intercept variance, an alternative GLM logistics model was applied to model the main outcome, which did not materially change the significance. Model diagnostics for the full data set can be found in Table S1. Model diagnostics for data sets excluding PACU and excluding “UTA” are found in Tables S2 and S3, respectively.

As seen in Table 2, there was no statistically significant difference in the odds of developing delirium in a room with a window in adjusted multivariate analysis (aOR 2.0 (95% CI 0.8–5.2), *p* = 0.155) with an adjustment in Model 1 for room type, APACHE IV mortality risk score, diagnosis, and ICU type, and (aOR 2.2 (95% CI 0.7–6.2), *p* = 0.155) in Model 2 after adjustment for room type, APACHE IV mortality risk score, diagnosis, ICU type, MV, sedation, and restraints.

### 3.4. Secondary Outcomes

#### 3.4.1. Length of Stay, Disposition and Mortality

No missingness was encountered in the multivariate gamma mixed model. To assess the model fitting of the primary gamma mixed model on hospital LOS, simulation-based residual diagnostics (DHARMa) showed no significant evidence of outliers, although mild overdispersion was observed (dispersion ≈ 1.32). Regarding ICU LOS, model diagnostics were performed using simulation-based residuals (DHARMa), which did not indicate major deviations from model assumptions, although moderate overdispersion (dispersion ≈ 1.70) was observed (Tables S4 and S5). For hospital LOS, the room-level random-intercept variance was estimated to be essentially zero, and the gamma mixed model showed a singular fit, so a simpler gamma generalized linear model was used. For ICU LOS, the estimated room-level variance was small but nonzero, and the gamma mixed model did not show singularity. Therefore, the gamma mixed-effects model was retained for that outcome.

In terms of the in-hospital death and ICU mortality outcomes, the logistic mixed-effects models showed singular fits because the room-level random intercept variance was estimated to be essentially zero. Therefore, as an alternative approach, we fitted logistic regression models without a random intercept and applied room-level cluster-robust standard errors.

Hospital LOS was longer for patients in a room with a window, even in adjusted analyses (Model 1: mean ratio (MR) 1.4 (95% CI 1.1–1.8; *p* = 0.008); Model 2: MR 1.4 (95% CI 1.1–1.8; *p* =0.008). Because multiple secondary comparisons were examined, however, we applied FDR correction, and the association no longer remained statistically significant (Table S6). No significant difference was evident for patients with closed-format compared to open-format rooms in Model 1 adjusted analyses, MR 0.8 (95% CI 0.5–1.3; *p* = 0.378) or in Model 2 adjusted analyses, MR 0.8 (95% CI 0.5–1.2, *p* = 0.267).

There was no significant difference in ICU LOS in either adjusted model for rooms with windows compared to no windows. For patients in closed- versus open-format rooms, there was no significant difference in ICU LOS. Likewise, regarding disposition and risk of mortality, there were no significant differences observed based on room design. Length of stay, disposition, and mortality outcomes are presented in Table 3.

#### 3.4.2. Exploratory Outcome: Composite Incidence of CAM-ICU Positive Plus CAM-ICU UTA

Two hundred four (204) patients experienced this composite score (44.5%) with 23 (31.9%) and 181 (46.9%), in windowless or windowed rooms, 44 (39.3%) in open-format rooms, and 160 (46.2%) in rooms with doors, respectively. The multivariate analyses did not show a significant difference between this composite score and room type, as shown in Table 2.

### 3.5. Sensitivity Analyses

We performed sensitivity analyses with both exclusion of PACU as well as exclusion of CAM-ICU UTA observations and repeated both primary and multivariable models. The results of these analyses were generally consistent with those from the full dataset. Specifically, no associations between room characteristics and delirium outcomes reached statistical significance after FDR correction, and the direction and magnitude of effect estimates remained similar (Tables S7 and S8).

## 4. Discussion

We hypothesized that patients in windowless or open-format rooms would have a higher incidence of delirium, defined by a positive CAM-ICU score. Our study did not show an association between the primary outcome of CAM-ICU positive status with windowless rooms or rooms without doors. Furthermore, neither the presence of a window nor room format was significantly associated with in-hospital or ICU mortality. There was an association between the presence of windows and increased hospital LOS in both multivariate models; however, after FDR correction, the statistical significance was no longer present.

These findings contrast and support some recent findings by Anderson et al., who demonstrated a surprising association of an increased odds of developing delirium in patients in windowed rooms [20]. In fact, in their study, patients in rooms with windows had an increased time with delirium, as well as non-significant increases in ICU and hospital LOS. This challenges the current belief that windows in the ICU are exclusively beneficial and stimulates discussion regarding other factors that might be contributory. There are some important differences between these studies. In contrast to our medical cohort, Anderson et al. evaluated a cohort of 3527 patients in the surgical ICU but only evaluated presence or absence of window, compared to our study that evaluated various room factors. Similarly to our study, patient acuity scores were slightly higher in patients in rooms with windows. By focusing on a medical population and incorporating other granular information such as whether there was a view of the window from the bed, our study attempts to provide a complementary perspective on architectural influencers on ICU outcomes. In 34% of our rooms, there was no direct view of the window, likely also affecting patient exposure to natural light. A window located behind a bed may still provide enough ambient light to assist with circadian rhythms. On the other hand, if a patient is unable to see outside and have a visual orientation to the world, they may not have a robust benefit from a window. In addition, whether window coverings are utilized may attenuate the effects of a window as well. These aspects of windows in the ICU are not fully understood, and there are mixed studies on whether natural light affects the development of delirium [16,21,22]. One prospective study, which found no reduction in delirium when comparing rooms with or without windows, interestingly found that natural light exposure is associated with reduced risk of agitation and hallucinations [23]. Inconsistent findings can make it challenging to establish clear guidelines and associations.

The effects of other environmental factors may affect the incidence of delirium as well. Studies looking at multi-patient compared to single-patient rooms demonstrate a decreased prevalence or number of days with delirium in patients in single-bed rooms [2,17]. Our study utilized the description of rooms with doors or without doors as a surrogate for single-bed or multi-bed rooms, respectively. These different room configurations have different sound and light exposures [17,24]. Elevated and excessive noise levels are an important contributor to ICU environments which can tend to disturb sleep [25,26]. Guidelines suggest optimizing the environment for the reduction of noise and possibly the use of sleep aids like masks and ear plugs to reduce light and noise [9,27]. Closed rooms, however, would be expected to have lower levels of external noise. Our findings demonstrated no association with delirium in these rooms. One non-ICU study that looked at patients’ distance to ward entrances found a higher incidence of critical illness and increased LOS in patients further from the entrance [28]. This may demonstrate that decreased attention and delays in care are associated with distance to the bed. Our study did not evaluate distance to nursing stations and other environmental factors associated with room types. These factors should be evaluated in future larger studies to assess potential confounding variables.

Our study has several limitations. This was a single-center study of adults in an urban tertiary care center, and we excluded several patient populations, including surgical patients. This could contribute to a decreased ability to generalize our results to other patient populations. Additionally, we excluded 42 patients who underwent room transfers during their intensive care stay. This exclusion may have introduced a degree of selection bias, as the necessity to change rooms often indicates shifts in a patient’s acuity, the need for specialized isolation, or fluctuating bed availability within the unit. By omitting this group, we may have excluded individuals with a different risk profile for delirium, which could have influenced our ability to detect a significant association.

The retrospective and non-randomized nature of our study is also a limitation. We attempted to restrict confounders by including a sample of patients that were cared for by one team of medical intensivists, decreasing variability in management. Because ICU beds are a scarce resource, randomization in this type of study is limited because effectively randomizing patients to specific room types could inhibit providing for patients’ critical needs and risks delaying care by waiting for specific bed types to become available. Our study included patients across geographical units with different rates of windows and doors. Yet, ICU room characteristics were not randomly distributed across ICU departments, and the exposure variables remained partially confounded with unit-level workflow and care culture. Furthermore, patients in windowed rooms had higher illness severity and greater use of interventions such as mechanical ventilation and sedation. Our secondary models adjusted for mechanical ventilation, sedation exposure, and restraint use as proxies for patient acuity and clinical complexity. Residual confounding, however, cannot be fully excluded, particularly given the structural alignment between room characteristics and ICU unit type; therefore, the observed associations should be interpreted as contextual/environmental associations rather than fully isolated architectural effects.

Another limitation is that some combinations of room characteristics, for example, “no window and no door”, were rare, so the study was not well powered to evaluate specific room-configuration subgroups, despite no significant detection of window-by-door interaction in our analysis. Similarly, subgroups of patients in rooms with specific configurations, such as those with partially obstructed windows or windows located behind the bed, were too small for independent statistical evaluation. Consequently, our binary classification of rooms was relatively simplified; it did not account for whether a window was within the patient’s direct line of sight or if it was functionally irrelevant for circadian entrainment. These exposures are likely clinically relevant and warrant further investigation in larger, multi-center studies that move beyond architectural presence to include objective measures of noise and ambient natural light levels. Our sample sizes were limited to a specific retrospective timeframe, and we could not perform further collection of data beyond the study period due to system-wide changes in the methods of CAM-ICU obtainment that would confound our results.

In order to reduce limitations due to inaccurately obtained CAM-ICU scores, we provided education regarding CAM-ICU documentation prior to the study period in hopes of increasing documentation and reducing missed delirium diagnoses; however, true delirium may still be missed due to systemic issues such as time of day, location in the unit, and use of restraints or sedation [29,30]. One factor that can easily affect performance of CAM-ICU scoring and thereby affects the detection of delirium is oversedation. The absence of RASS data is a notable limitation of this study, and we cannot definitively rule out whether room characteristics influenced sedation practices and CAM-ICU assessments rather than delirium incidence alone. Despite this, our facility did have a nurse-directed daily sedation vacation protocol in effect during the study period, which would represent a systematic effort to minimize sedation and minimize this limitation. We included CAM-ICU positive plus CAM-ICU UTA as an exploratory outcome to consider possible hypoactive delirium; yet, lack of information regarding sedation depth at the time of CAM-ICU assessment inhibited our ability to account for time-at-risk or whether our CAM-ICU UTA was due to oversedation, unrecognized delirium, or other comatose state. In other words, a patient labeled as “CAM-ICU UTA” may represent distinct clinical states, and conflating these conditions may hinder diagnostic clarity rather than enhance it. Moreover, the sensitivity of this study may be limited by the twice-daily CAM-ICU assessment protocol, as more frequent monitoring may be required to capture the fluctuating nature of delirium. This, combined with the absence of granular RASS data, represents a key limitation in our ability to fully evaluate the environmental impact on patient mental status.

Lastly, our findings that room characteristics were not associated with delirium must be interpreted in the context of limited statistical power. With the substantial difference in group sizes between windowed and non-windowed rooms, the study was likely underpowered to reach statistical significance for the primary outcome. The lack of statistical significance should be viewed as inconclusive rather than definitive evidence of no effect of windows on the development of delirium in the ICU.

## 5. Conclusions

In this study, the presence of windows and closed-format rooms was not significantly associated with the incidence of delirium. While initial multivariate analysis suggested an association between windowed rooms and increased hospital LOS, this finding did not remain statistically significant after FDR correction. These findings must be interpreted cautiously; due to unequal group sizes, the study was likely underpowered to detect moderate differences in delirium outcomes. Nevertheless, this research contributes to the expanding literature exploring how ICU architectural factors may influence clinical outcomes.

## Figures and Tables

**Table 1:** Patient demographic and clinical characteristics compared to room characteristics.

Characteristic	Total, *n* = 458	No Window, *n* = 72	Window, *n* = 386	Window Difference, *p*	Open Format, *n* = 112	Closed Format, *n* = 346	Format Difference, *p*
Age, years Mean (SD)	61.7 (16.4)	62.2 (16.5)	61.6 (16.4)	0.765	61.7 (17.1)	61.6 (16.2)	0.969
Male, *n* (%)	235 (51.3)	29 (40.3)	206 (53.4)	0.056	50 (44.6)	185 (53.5)	0.130
Race, *n* (%) White Black Other	352 (76.9) 84 (18.3) 22 (4.8)	49 (68.1) 19 (26.4) 4 (5.6)	303 (78.5) 65 (16.8) 18 (4.7)	0.13 *	86 (76.8) 22 (19.8) 4 (3.6)	266 (76.9) 62 (17.9) 18 (5.2)	0.798 *
Language, *n* (%) English Spanish Other	414 (90.4) 40 (8.7) 4 (0.9)	65 (90.3) 7 (9.7) 0 (0)	349 (90.4) 33 (8.5) 4 (1)	0.91 *	105 (93.8) 5 (4.5) 2 (1.8)	309 (89.3) 35 (10.1) 2 (0.6)	0.075 *
Diagnosis, *n* (%) Sepsis Respiratory Cardiac GI Other	74 (16.2) 143 (31) 71 (15.5) 41 (9) 129 (28.2)	13 (18.1) 19 (26.4) 13 (18.1) 6 (8.3) 21 (29.2)	61 (15.8) 124 (32.1) 58 (15) 35 (9.1) 108 (28.0)	0.869	13 (11.6) 37 (33.0) 16 (14.4) 6 (5.4) 40 (35.7)	61 (17.6) 106 (30.6) 55 (15.9) 35 (10.1) 89 (25.7)	0.119
APACHE IV Predicted Mortality **, Median (IQR)	10.4 (4.6, 27.2)	8.3 (2.6, 19.9)	11.1 (5, 29.8)	0.022	8.2 (3.1, 21.9)	11.4 (5.2, 30.2)	0.015
MV, *n* (%)	146 (31.9)	14 (19.4)	132 (34.2)	0.02	27 (24.1)	119 (34.4)	0.056
Restraint, *n* (%)	108 (23.6)	12 (16.7)	96 (24.9)	0.176	22 (19.6)	86 (24.9)	0.317
Sedation, *n* (%)	165 (36)	18 (25)	147 (38.1)	0.047	33 (29.5)	132 (38.2)	0.121
Home Antipsychotic, *n* (%)	31 (6.8)	4 (5.6)	27 (7)	0.802 *	6 (5.4)	25 (7.2)	0.64
Time Intubated, Days, Median (IQR)	0 (0,1.3)	0 (0,0)	0 (0, 1.5)	0.009	0 (0, 0)	0 (0, 1.5)	0.034
Time Restrained, Days, Median (IQR)	0 (0, 0)	0 (0, 0)	0 (0, 0)	0.111	0 (0, 0)	0 (0, 0)	0.215

SD = Standard deviation; GI = gastrointestinal; APACHE IV = Acute Physiology and Chronic Health Evaluation; MV = mechanical ventilation; IQR = Interquartile Range. * Represents Fisher Exact Test due to small cell counts. ** APACHE IV Predicted mortality represents a statistically derived probability of in-hospital death based on admission physiology. Continuous variables summarized as mean (SD) were compared using Student’s *t*-test; continuous variables summarized as median (IQR) were compared using the Wilcoxon rank-sum test; categorical variables were compared using Pearson’s chi-square test unless otherwise indicated by * for Fisher’s exact test.

**Table 2:** Primary outcome of Confusional Assessment Method for the ICU (CAM-ICU) positive and exploratory outcome related to window and room format with multivariate regression analysis.

	Exposure No	Exposure Yes	Multivariate Primary Model ^1^		Multivariate Secondary Model ^2^	
			Estimate * (95% CI)	*p*	Estimate * (95% CI)	*p*
CAM-ICU positive, *n* (%)						
Window Present	14 (19.4)	102 (26.4)	2 (0.8, 5.2)	0.155	2.2 (0.7, 6.2)	0.155
Closed Format	32 (28.6)	84 (24.3)	0.3 (0.1, 2.2)	0.251	0.1 (0, 1.2)	0.077
CAM-ICU positive + UTA, *n* (%)						
Window Present	23 (31.9)	181 (46.9)	1.6 (0.6, 4.6)	0.376	1.4 (0.4, 4.5)	0.597
Closed Format	44 (39.3)	160 (46.2)	0.6 (0.1, 4.2)	0.642	0.3 (0, 2.4)	0.24

Multivariate Primary Model ^1^ includes Windowed Room, Closed Format, APACHE IV mortality risk Score (cubic spline), Diagnosis, and ICU type as fixed effects and Room as a random intercept. Multivariate Secondary Model ^2^ includes Windowed Room, Closed Format, APACHE IV mortality risk Score (cubic spline), MV, Sedation, Restraint, Diagnosis, and ICU type as fixed effects and Room as a random intercept. * Estimates for binary outcomes are odds ratios of windowed vs. no window, closed format vs. open format estimated by the logistic mixed models. CAM-ICU = Confusional Assessment Method for the ICU; CI = Confidence Interval; UTA = unable to assess.

**Table 3:** Secondary outcomes related to window and room format with multivariate regression analysis.

	Exposure No	Exposure Yes	Multivariate Primary Model ^1^		Multivariate Secondary Model ^2^	
			Estimate * (95% CI)	*p*	Estimate * (95% CI)	*p*
Disposition, *n* (%) In-hospital Death						
Window Present	8 (11.1)	63 (16.3)	1.1 (0.5, 2.3)	0.885	0.9 (0.4, 2.3)	0.881
Closed Format	13 (11.6)	58 (16.8)	2.3 (0.9, 5.7)	0.074	2.1 (0.8, 5.5)	0.145
ICU Mortality, *n* (%)						
Window Present	8 (11.1)	56 (14.5)	1 (0.4, 2.2)	0.974	0.9 (0.3, 2.4)	0.757
Closed Format	13 (11.6)	51 (14.7)	2.3 (0.9, 5.9)	0.078	2.1 (0.7, 5.8)	0.164
ICU LOS, days Median (IQR)						
Window Present	2.1 (1.7, 3.7)	2.3 (1.6, 4.6)	1.3 (1, 1.7)	0.086	1.2 (0.9, 1.7)	0.131
Closed Format	2.2 (1.8, 3.9)	2.3 (1.6, 4.6)	1.2 (0.7, 2)	0.564	1 (0.6, 1.7)	0.907
Hospital LOS, days Median (IQR)						
Window Present	5.7 (3.5, 9.3)	7.2 (4.1, 12.5)	1.4 (1.1, 1.8)	0.008	1.4 (1.1, 1.8)	0.008
Closed Format	5.9 (3.7, 10.3)	7.2 (4.1, 12.4)	0.8 (0.5, 1.3)	0.378	0.8 (0.5, 1.2)	0.267

Multivariate Primary Model ^1^ includes Windowed Room, Closed Format, APACHE IV mortality risk Score (cubic spline), Diagnosis, and ICU type. Multivariate Secondary Model ^2^ includes Windowed Room, Closed Format, APACHE IV mortality risk Score (cubic spline), MV, Sedation, Restraint, Diagnosis, and ICU type. The In-hospital Death and ICU mortality outcomes were fitted with logistic regression models due to singular fit, with room-level cluster-robust standard error adjustment to generate 95% CI and *p* values. Estimates for ICU LOS outcomes are mean ratios of window vs. no window, closed format vs. open format estimated by the gamma mixed models. The Hospital LOS was fitted with gamma regression models due to singular fit, to generate 95% CI and *p* values. * Estimates for binary outcomes are odds ratios of windowed vs. no window, closed format vs. open format estimated by the logistic mixed models. CI = Confidence Interval; ICU = Intensive Care Unit; LOS = Length of Stay.

## Data Availability

The datasets presented in this article are not readily available because the data were only available due to time limitations set by the IRB. Requests to access the datasets should be directed to the corresponding author.

## Supplementary Materials

The following supporting information can be downloaded at: https://www.mdpi.com/article/10.3390/neurosci7030061/s1, Table S1: Model Diagnostic table on the full data set; Table S2: Model Diagnostic table on the data set exclude PACU; Table S3: Model Diagnostic table on the data set exclude UTA samples; Table S4: Gamma fitting vs Negative Binomial fitting on Hospital LOS of all samples; Table S5: Gamma fitting vs Negative Binomial fitting on ICU LOS of all samples; Table S6: GLM model results with FDR correction on all data samples; Table S7: GLM models results on data excluding PACU; Table S8: GLM models results on data excluding UTA samples.

## Abbreviations

The following abbreviations are used in this manuscript:

APACHE	Acute Physiology and Chronic Health Evaluation
CAM-ICU	Confusion Assessment Method for the Intensive Care Unit
CI	Confidence Interval
FDR	False Discovery Rate
ICU	Intensive Care Unit
IQR	Interquartile Range
LOS	Length of Stay
MR	Mean Ratio
MV	Mechanical Ventilation
OR	Odds Ratio
PADIS	Pain, Agitation/Sedation, Delirium, Immobility, and Sleep disruption
RASS	Richmond Agitation Sedation Scale
UTA	Unable to Assess

## Appendix A

### Appendix A.1

**Table A1:** Room characteristics demonstrating the presence of window, open or closed format, whether there was a view of the window from a bed, and whether the room was near loud, automatic doors.

Dept.	Room Number	Closed Format	Windowed	View of Window from Bed	Room Near Automatic Door
ICU	1	Yes	Yes	Yes	Yes
ICU	2	Yes	Yes	Yes	No
ICU	3	Yes	Yes	Yes	No
ICU	4	Yes	Yes	Yes	No
ICU	5	Yes	Yes	Yes	No
ICU	6	Yes	Yes	Yes	No
ICU	7	Yes	Yes	Yes	No
ICU	8	Yes	Yes	Yes	No
ICU	9	Yes	Yes	Yes	No
ICU	10	Yes	Yes	Yes	No
ICU	11	Yes	Yes	Yes	No
ICU	12	Yes	Yes	Yes	No
ICU	13	Yes	Yes	Yes	No
ICU	14	Yes	Yes	Yes	No
ICU	15	Yes	Yes	Yes	No
ICU	16	Yes	Yes	Yes	No
CVICU	1	Yes	Yes	Yes	Yes
CVICU	2	Yes	Yes	Yes	No
CVICU	3	Yes	Yes	Yes	No
CVICU	4	Yes	Yes	Yes	No
CVICU	5	Yes	Yes	Yes	No
CVICU	6	Yes	Yes	Yes	No
CVICU	7	Yes	No	No	No
CVICU	8	Yes	Yes	No	No
CVICU	9	Yes	Yes	Yes	No
CVICU	10	Yes	Yes	Yes	No
CVICU	11	Yes	Yes	Yes	No
CVICU	12	Yes	Yes	Yes	No
CVICU	13	Yes	Yes	Yes	No
CVICU	14	Yes	Yes	Yes	No
CVICU	15	Yes	Yes	Yes	Yes
MSICU	1	No	No	No	Yes
MSICU	2	No	No	No	No
MSICU	3	No	No	No	No
MSICU	4	No	Yes	No	Yes
MSICU	5	No	Yes	No	No
MSICU	6	No	Yes	No	No
MSICU	7	No	Yes	No	No
MSICU	8	Yes	Yes	No	No
PACU	1	No	No	No	No
PACU	2	No	No	No	No
PACU	3	No	No	No	No
PACU	4	No	No	No	No
PACU	5	No	No	No	No
