# Supplementary material for: Intensive Care Unit Room Characteristics and Association with the Development of Delirium
Source: NeuroSci. 2026 May 20;7(3):61. doi: 10.3390/neurosci7030061 (PMC13214964; doi:10.3390/neurosci7030061)
Supplement: Supplementary file 1 [file neurosci-07-00061-s001.zip › neurosci-4273798-supplementary.pdf]

Table S1. Model Diagnostic table on the full data set.

Outcome	Model	Model Type	N	AIC	Random Intercept Variance	DHARMa Dispersion Ratio	Singular Fit	Convergence issue
CAM+ binary	Primary	Logistic mixed model	458	480.4	0.14		FALSE	None
CAM+ binary	Secondary	Logistic mixed model	458	427.7	0.16		FALSE	None
CAM+_UTA	Primary	Logistic mixed model	458	468.1	0.21		FALSE	None
CAM+_UTA	Secondary	Logistic mixed model	458	347.1	0.20		FALSE	None
HOSP mortality	Primary	Logistic mixed model	458	354.4	0.00		TRUE	boundary (singular) fit
HOSP mortality	Secondary	Logistic mixed model	458	338.6	0.00		TRUE	boundary (singular) fit
ICU mortality	Primary	Logistic mixed model	458	335.8	0.00		TRUE	boundary (singular) fit
ICU mortality	Secondary	Logistic mixed model	458	319.4	0.00		TRUE	boundary (singular) fit
ICU LOS	Primary	Gamma mixed model	458	1978.7	0.02	1.71	FALSE	None
ICU LOS	Secondary	Gamma mixed model	458	1881.8	0.03	1.61	FALSE	None
Hosp LOS	Primary	Gamma mixed model	458	2775.4	0.00	1.33	TRUE	None
Hosp LOS	Secondary	Gamma mixed model	458	2765.3	0.00	1.31	TRUE	None

Table S2. Model Diagnostic table on the data set exclude PACU.

Outcome	Model	Model Type	N	AIC	Random Intercept Variance	DHARMa Dispersion Ratio	Singular Fit	Convergence issue
CAM+ binary	Primary	Logistic mixed model	440	470.4	0.14		FALSE	None
CAM+ binary	Secondary	Logistic mixed model	440	420.8	0.16		FALSE	None
CAM+_UTA	Primary	Logistic mixed model	440	450.4	0.23		FALSE	None
CAM+_UTA	Secondary	Logistic mixed model	440	332.8	0.23		FALSE	None
HOSP mortality	Primary	Logistic mixed model	440	347.4	0.00		TRUE	boundary (singular) fit
HOSP mortality	Secondary	Logistic mixed model	440	330.7	0.00		TRUE	boundary (singular) fit

ICU mortality	Primary	Logistic mixed model	440	328.6	0.00		TRUE	boundary (singular) fit
ICU mortality	Secondary	Logistic mixed model	440	311.2	0.00		TRUE	boundary (singular) fit
ICU LOS	Primary	Gamma mixed model	440	1916.4	0.02	1.68	FALSE	None
ICU LOS	Secondary	Gamma mixed model	440	1822.6	0.03	1.59	FALSE	None
Hosp LOS	Primary	Gamma mixed model	440	2683.0	0.00	1.32	TRUE	None
Hosp LOS	Secondary	Gamma mixed model	440	2672.6	0.00	1.30	TRUE	None

Table S3. Model Diagnostic table on the data set exclude UTA samples.

Outcome	Model	Model Type	N	AIC	Random Intercept Variance	DHARMa Dispersion Ratio	Singular Fit	Convergence issue
CAM+ binary	Primary	Logistic mixed model	370	349.1	0.49		FALSE	Model failed to converge
CAM+ binary	Secondary	Logistic mixed model	370	261.9	0.62		FALSE	None
CAM+_UTA	Primary	Logistic mixed model	370	349.1	0.49		FALSE	Model failed to converge
CAM+_UTA	Secondary	Logistic mixed model	370	261.9	0.62		FALSE	None
HOSP mortality	Primary	Logistic mixed model	370	235.8	0.00		TRUE	boundary (singular) fit
HOSP mortality	Secondary	Logistic mixed model	370	221.0	0.00		TRUE	boundary (singular) fit
ICU mortality	Primary	Logistic mixed model	370	212.3	0.00		TRUE	boundary (singular) fit
ICU mortality	Secondary	Logistic mixed model	370	196.9	0.00		TRUE	boundary (singular) fit
ICU LOS	Primary	Gamma mixed model	370	1562.5	0.02	1.87	FALSE	None
ICU LOS	Secondary	Gamma mixed model	370	1454.4	0.03	1.69	FALSE	None
Hosp LOS	Primary	Gamma mixed model	370	2234.7	0.00	1.34	TRUE	None
Hosp LOS	Secondary	Gamma mixed model	370	2220.9	0.00	1.31	TRUE	None

Table S4. Gamma fitting vs Negative Binomial fitting on Hospital LOS of all samples.

Model_Name	Model_Type	DHARMa Dispersion Ratio	DHARMa Dispersion Pvalue	DHARMa Outlier Pvalue	AIC	BIC	logLik
Primary	Gamma GLM	1.317	0.012	0.585	2682.2	2735.3	-1328.1
Primary	Negative Binomial	1.323	0.014	1	2716.2	2769.3	-1345.1

Exploratory	Gamma GLM	1.308	0.03	0.22	2671.7	2737.1	-1319.9
Exploratory	Negative Binomial	1.323	0.008	0.92	2707.0	2772.3	-1337.5

Table S5. Gamma fitting vs Negative Binomial fitting on ICU LOS of all samples.

Model_Name	Model_Type	DHARMa Dispersion Ratio	DHARMa Dispersion Pvalue	DHARMa Outlier Pvalue	AIC	BIC	logLik
Primary	Gamma GLM	1.745	<0.001	0.002	1920.7	1973.8	-947.3
Primary	Negative Binomial	1.489	<0.001	0.2	2038.0	2091.2	-1006.0
Exploratory	Gamma GLM	1.677	0.002	0.002	1830.6	1896.0	-899.3
Exploratory	Negative Binomial	1.445	<0.001	0.2	1965.2	2030.6	-966.6

Table S6. GLM model results with FDR correction on all data samples.

	Exposure No	Exposure Yes	Multivariate Primary Model[1]			Multivariate Secondary Model[2]		
			**Estimate* (95% CI)**	**p**	**BH FDR**	**Estimate* (95% CI)**	**p**	**BH FDR**
**CAM-ICU positive, n (%)**								
Window	14 (19.4)	102(26.4)	1.9 (0.8, 4.4)	0.119	0.425	2.1 (0.8, 5.1)	0.124	0.425
Closed Format	32 (28.6)	84 (24.3)	0.3 (0.1, 1.8)	0.212	0.565	0.2 (0, 1)	0.055	0.425
**CAM-ICU positive + UTA, n (%)**								
Window	23 (31.9)	181(46.9)	1.5 (0.7, 3.6)	0.33	0.647	1.3 (0.5, 3.7)	0.6	0.758
Closed Format	44 (39.3)	160(46.2)	0.6 (0.1, 3.1)	0.579	0.758	0.3 (0, 1.8)	0.179	0.536
**Disposition, In-hospital Death n (%)**								
Window	8 (11.1)	63 (16.3)	1.1 (0.4, 3)	0.915	0.955	0.9 (0.3, 2.8)	0.902	0.955
Closed Format	13 (11.6)	58 (16.8)	2.3 (0.4,13.9)	0.368	0.647	2.1 (0.3, 14)	0.459	0.689
**ICU Mortality, n (%)**								
Window	8 (11.1)	56 (14.5)	1 (0.3, 2.9)	0.98	0.98	0.9 (0.3, 2.7)	0.78	0.936
Closed Format	13 (11.6)	51 (14.7)	2.3 (0.4, 14)	0.361	0.647	2.1 (0.3, 14)	0.455	0.689
**ICU LOS, days Median (IQR)**								
Window	2.1 (1.7, 3.7)	2.3 (1.6,4.6)	1.3 (1, 1.7)	0.094	0.425	1.2 (0.9, 1.6)	0.119	0.425
Closed Format	2.2 (1.8, 3.9)	2.3 (1.6,4.6)	1.2 (0.7, 2)	0.599	0.758	1 (0.6, 1.7)	0.889	0.955
**Hospital LOS, days Median (IQR)**								
Window	5.7 (3.5, 9.3)	7.2 (4.1, 12.5)	1.4 (1.1, 1.8)	0.008	0.1	1.4 (1.1, 1.8)	0.008	0.1
Closed Format	5.9 (3.7, 10.3)	7.2 (4.1, 12.4)	0.8 (0.5, 1.3)	0.378	0.647	0.8 (0.5, 1.2)	0.267	0.64

Table S7. GLM models results on data excluding PACU.

	Exposure No	Exposure Yes	Multivariate Primary Model[1]			Multivariate Secondary Model[2]		
			Estimate* (95% CI)	p	BH FDR	Estimate* (95% CI)	p	BH FDR
**CAM-ICU positive, n (%)**								
Window	13 (24.1)	102 (26.4)	1.9 (0.8, 4.4)	0.118	0.434	2 (0.8, 5.1)	0.124	0.434
Closed Format	31 (33)	84 (24.3)	0.3 (0.1, 1.9)	0.215	0.574	0.2 (0, 1.1)	0.062	0.434
**CAM-ICU positive + UTA, n (%)**								
Window	21 (38.9)	181 (46.9)	1.5 (0.7, 3.7)	0.325	0.674	1.3 (0.5, 3.8)	0.592	0.777
Closed Format	42 (44.7)	160 (46.2)	0.6 (0.1, 3.1)	0.575	0.777	0.3 (0, 1.8)	0.179	0.536
**Disposition, n (%) In-hospital Death**								
Window	7 (13)	63 (16.3)	1.1 (0.4, 3)	0.923	0.963	0.9 (0.3, 2.8)	0.888	0.963
Closed Format	12 (12.8)	58 (16.8)	2.3 (0.4, 13.7)	0.372	0.674	2 (0.3, 13.8)	0.463	0.694
**ICU Mortality, n (%)**								
Window	7 (13)	56 (14.5)	1 (0.3, 2.9)	0.972	0.972	0.8 (0.3, 2.6)	0.765	0.919
Closed Format	12 (12.8)	51 (14.7)	2.3 (0.4, 13.8)	0.366	0.674	2.1 (0.3, 13.8)	0.459	0.694
**ICU LOS, days Median (IQR)**								
Window	2.1 (1.6, 3.8)	2.3 (1.6, 4.6)	1.3 (1, 1.7)	0.101	0.434	1.2 (0.9, 1.6)	0.127	0.434
Closed Format	2.2 (1.8, 4.1)	2.3 (1.6, 4.6)	1.2 (0.7, 2)	0.615	0.777	1 (0.6, 1.7)	0.904	0.963
**Hospital LOS, days Median (IQR)**								
Window	5.9 (3.8, 9.3)	7.2 (4.1, 12.5)	1.4 (1.1, 1.8)	0.009	0.106	1.4 (1.1, 1.8)	0.008	0.106
Closed Format	6.5 (3.9, 10.9)	7.2 (4.1, 12.4)	0.8 (0.5, 1.3)	0.393	0.674	0.8 (0.5, 1.2)	0.277	0.665

Table S8. GLM models results on data excluding UTA samples.

	Exposure No	Exposure Yes	Multivariate Primary Model[1]			Multivariate Secondary Model[2]		
			Estimate* (95% CI)	p	BH FDR	Estimate* (95% CI)	p	BH FDR
**CAM-ICU positive, n (%)**								
Window	14 (22.2)	102 (33.2)	1.4 (0.5, 3.8)	0.462	0.691	1.5 (0.5, 5.2)	0.485	0.691
Closed Format	32 (32)	84 (31.1)	0.2 (0, 1.6)	0.139	0.553	0.1 (0, 0.9)	0.045	0.268
**Disposition, n (%) In-hospital Death**								
Window	5 (7.9)	34 (11.1)	1.4 (0.4, 5.1)	0.649	0.76	1.7 (0.4, 7.1)	0.467	0.691
Closed Format	11 (11)	28 (10.4)	2 (0.3, 13.1)	0.477	0.691	1.6 (0.2, 11.7)	0.665	0.76
**ICU Mortality, n (%)**								
Window	5 (7.9)	28 (9.1)	1.3 (0.3, 5)	0.708	0.76	1.6 (0.4, 6.9)	0.523	0.697
Closed Format	11 (11)	22 (8.1)	1.9 (0.3, 12.9)	0.49	0.691	1.4 (0.2, 11)	0.728	0.76
**ICU LOS, days Median (IQR)**								
Window	2 (1.7, 3.5)	2.2 (1.6, 3.9)	1.2 (0.9, 1.6)	0.287	0.691	1.2 (0.9, 1.6)	0.212	0.635

Closed Format	2.2 (1.7, 3.7)	2.2 (1.6, 3.9)	1.2 (0.6, 2.2)	0.607	0.76	1 (0.6, 1.7)	0.98	0.98
**Hospital LOS, days Median (IQR)**								
Window	5.8 (3.7, 9.2)	7.3 (4.1, 13)	1.4 (1.1, 1.8)	0.014	0.167	1.4 (1.1, 1.8)	0.009	0.167
Closed Format	6.1 (3.7, 10.8)	7.3 (4, 12.7)	0.8 (0.4, 1.3)	0.326	0.691	0.7 (0.4, 1.2)	0.161	0.553
